# Reproductive risk factors in breast cancer and genetic hormonal pathways: a gene-environment interaction in the MCC-Spain project

**DOI:** 10.1186/s12885-018-4182-3

**Published:** 2018-03-12

**Authors:** Trinidad Dierssen-Sotos, Camilo Palazuelos-Calderón, José-Juan Jiménez-Moleón, Nuria Aragonés, Jone M. Altzibar, Gemma Castaño-Vinyals, Vicente Martín-Sanchez, Inés Gómez-Acebo, Marcela Guevara, Adonina Tardón, Beatriz Pérez-Gómez, Pilar Amiano, Victor Moreno, Antonio J. Molina, Jéssica Alonso-Molero, Conchi Moreno-Iribas, Manolis Kogevinas, Marina Pollán, Javier Llorca

**Affiliations:** 1 Universidad de Cantabria – IDIVAL, Santander, Spain; 2 CIBER Epidemiología y Salud Pública (CIBERESP), Madrid, Spain; 3 Universidad de Granada – ibs.Granada, Granada, Spain; 4 Cancer and Environmental Epidemiology Unit, National Center for Epidemiology, Carlos III Institute of Health, Avenida Monforte de Lemos 5, 28029 Madrid, Spain; 5 Cancer Epidemiology Research Group, Oncology and Hematology Area, IIS Puerta de Hierro (IDIPHIM), Manuel de Falla 1, 28222 Madrid, Spain; 6 Breast Cancer Early Detection Programme, Basque Health Service-Osakidetza, San Sebastian, Spain; 7 ISGlobal, Centre for Research in Environmental Epidemiology (CREAL), Barcelona, Spain; 8 IMIM (Hospital del Mar Medical Research Institute), Barcelona, Spain; 9 Universitat Pompeu Fabra (UPF), Barcelona, Spain; 10 Universidad de León, León, Spain; 11 Public Health Institute of Navarra, IdiSNA, Pamplona, Spain; 12 IUOPA, Universidad de Oviedo, Asturias, Spain; 13 Public Health Division of Gipuzkoa, BioDonostia Research Health Institute, San Sebastian, Spain; 14 IDIBELL-Catalan Institute of Oncology, L’Hospitalet de Llobregat, Barcelona, Spain; 15 Department of Clinical Sciences, Faculty of Medicine, University of Barcelona, Barcelona, Spain; 16 Health Services Research on Chronic Patients Network, REDISSEC, Valencia, Spain; 17 Facultad de Medicina, Universidad de Cantabria, Avda. Herrera Oria s/n, 39011 Santander, Spain

**Keywords:** Breast cancer, Genetic interactions, Reproductive factors

## Abstract

**Background:**

Reproductive factors are well known risk factors for breast cancer; however, little is known about how genetic variants in hormonal pathways interact with that relationship.

**Methods:**

One thousand one hundred thirty nine cases of breast cancer in women and 1322 frequency-matched controls were compared. Genetic variants in hormonal pathways (identified in the Kyoto Encyclopedia of Genes and Genomes) were screened according to their relationship with breast cancer using the Cochran-Armitage statistic. Information on reproductive factors was obtained using a face-to-face questionnaire. The interaction among the selected genetic variants and reproductive factors was tested with logistic regression.

**Results:**

Concerning C allele in rs2229712, compared to nulliparity in non-carriers the ORs for 1–2 and > 2 deliveries were 0.48 (0.28–0.81) and 0.34 (0.19–0.59), and in C carriers they were 0.92 (0.42–1.98) and 0.71 (0.31–1.61). Similar results were found in women carrying the C allele in rs1269851. Carriers of Allele T in rs35652107 and allele C in rs6018027 had the delivery number effect more pronounced.

**Conclusions:**

The number of deliveries had a dose-response protective effect on breast cancer; women carrying C allele in rs2229712 did not benefit from this protective effect.

**Electronic supplementary material:**

The online version of this article (10.1186/s12885-018-4182-3) contains supplementary material, which is available to authorized users.

## Background

Breast cancer is the most frequent cancer in women with about 1.7 million new cases per year, and the fifth cause of death from cancer overall with about 0.5 million deaths per year [1].

Estrogen levels seem to play a major role in breast cancer [2]. Several reproductive factors, e.g. early age at menarche, no parity, later age at first birth and later age at menopause, have been identified as risk factors for breast cancer as they are associated with higher levels of estrogens [3].

Women having a first-degree relative diagnosed with breast cancer have twice the risk of being affected by this disease [4]. A few rare genetic variants with high or moderate penetrance in BRCA1, BRCA2, TP53, PTEN, STK11, ATM, CHEK2, BRIP1, RAD51C, RAD51D, BARD1 and PALB2 genes increase the risk for breast cancer [5]; additionally, about 90 more frequent variants with low penetrance have also been identified, mainly in genome-wide association studies (GWAS) [6]. Together these genetic variants explain about 37% of the excess familial risk [7]. Some studies have focused on gene-environment interactions [7], however the stringent requirements for *p* values in GWAS make it difficult to study genetic variants – environment interactions [8].

Attention has rarely been paid to breast cancer genetic variants and their interaction with reproductive factors. Several genetic pathways have been identified as related to sexual hormone production or signalling. To the best of our knowledge a systematic evaluation of their interaction with reproductive factors leading to breast cancer has not been conducted yet. The main objective in this paper was to study the effect of the interaction between genetic variants in hormonal related pathways and reproductive factors on breast cancer risk.

## Methods

### The MCC-Spain study

**Fig. 1:**
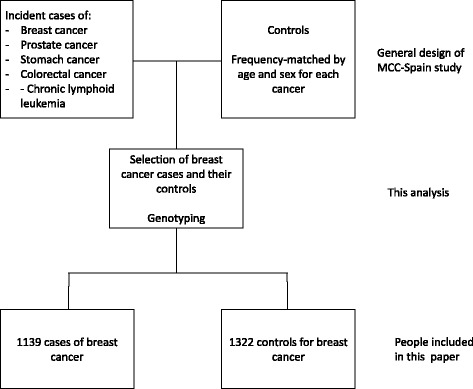
Recruitment procedure in the MCC-Spain study

The Multi Case-Control (MCC-Spain) study is a population-based case-control study of common tumours in Spain and has been described elsewhere [8]. It has been carried out in 12 Spanish provinces. Recruitment included incident cases of colorectal, breast, stomach and prostate cancer, and chronic lymphocytic leukemia diagnosed between September 1st, 2008 and December 31st, 2013, in patients aged between 20 and 85 years old, and resident within the catchment area of the hospital at least 6 months prior to recruitment; this report only refers to breast cancers and their controls (Fig. 1).

Cases were identified through an active search that included periodical visits to the collaborating hospital departments (i.e. gynaecology, oncology, general surgery, radiotherapy, and pathology departments), but only histologically confirmed incident cases of breast cancer (ICD-10: C50, D05.1, D05.7) with no prior history of the disease were included in this study. Ten out of 12 provinces recruited breast cancer cases and controls. Controls were selected from the general population according to age and sex distribution of the cases included in the study. In this paper, 1139 cases of breast cancer in women and 1322 frequency-matched controls were included.

Response rates were 71% for breast cancer and 53% for controls, with no differences in age and province of residence among those who participated and those who refused to participate. The Ethics Committees of participating hospitals approved the study protocols and participants provided written informed consent at the time of enrolment.

### Data collection

Participants were interviewed face-to-face by trained interviewers with a comprehensive epidemiological questionnaire that assessed socio-demographic information, personal and family history of cancer, anthropometric data, smoking habits, occupation, physical activity, water consumption, reproductive and medical history and medication/drugs use, sun exposure, sleep habits, use of hygiene products and cosmetics, signs and symptoms. Participant’s weight was recorded by self-report, as estimated 1 year before diagnosis for cases and for controls. Body mass index (BMI) was estimated from self-reported weight and height 1 year before the diagnosis for cases and 1 year prior to the interview for controls. Similar estimates provided total energy consumption.

Regarding reproductive history, detailed information was obtained on age at menarche, parity, age at first delivery, menopausal status, age at menopause, hormonal contraceptive use, and postmenopausal hormone therapy.

### Genotyping

The Infinium Human Exome BeadChip (Illumina, San Diego, USA) was used to genotype > 200,000 coding markers plus 5000 additional custom SNPs selected from previous GWAS studies or in genes of interest.

For this analysis we only considered the SNPs selected as follows. Firstly, we searched for sexual hormone pathways in the Kyoto Encyclopedia of Genes and Genomes (KEGG; http://www.genome.jp/kegg/) [9]; the following pathways were selected: hsa00140 (steroid hormone biosynthesis); hs4912 (GnRH signalling pathway); hsa04913 (ovarian steroidogenesis); hsa04914 (progesterone-mediated oocyte maturation); hsa04915 (estrogen signalling pathway); hsa04917 (prolactin signalling pathway); hsa04921 (oxytocin signalling pathway); hsa05213 (endometrial cancer); hsa05215 (prostate cancer); Additional file 1: Table S1 reports the genes included in each pathway and the number of SNPs in each gene that are included in the Infinium Human Exome BeadChip (See Additional file 1). Secondly, we performed a preliminary analysis (screening) with the SNPs included in these pathways in KEGGS (see statistical methods, below). Thirdly, the main analysis was carried out using only those SNPs that passed the screening.

### Statistical analysis

For each preselected SNP, the Cochran-Armitage statistics was estimated for the SNP-breast cancer (as a whole or any of its subtypes) relationship, using the metric 0, 1, 2 for the number of mutated alleles (i.e.: we assumed the additive model of inheritance). SNPs with *p* value lower than 0.001 were included in the main analysis.

In order to adjust for confounding factors, a propensity score was built using non-reproductive risk factors for breast cancer. In order to do that, we performed a logistic regression analysis with breast cancer as outcome and age at recruitment, province of residence, educational level, family history of breast cancer, smoking, and BMI as regressors; the propensity score was created as the predicted probability of being a breast cancer case according to this logistic regression.

The main analysis was carried out by logistic regression, using breast cancer as effect and reproductive factors as regressors. This analysis was performed twice: first, without genetic information; second, stratifying by each selected SNP. All logistic regression models were adjusted for the propensity score and the remaining reproductive factors. Their results are reported as odds ratios (OR) with 95% confidence intervals (CI). All statistical analyses were performed with the statistical package Stata 14/SE (Stata Corp. College Station, TX, USA).

## Results

### Sample description

One thousand one hundred thirty nine cases and 1322 controls were included in the analysis. Table 1 displays the main characteristics of the sample. Breast cancer cases were 2 years younger than controls on average (56.8 ± 12.6 years for cases and 59.2 ± 13.2 for controls) and they had a slightly lower educational level. Cases had a higher number of deliveries than controls, but they did not differ in age at menarche, age at first birth or age at menopause. Eight hundred forty nine tumors were hormone (estrogen or progesterone) receptors positive, 161 were ERBB2 positive and 102 were triple negative.

**Table 1:** Sample description

Variable	Category	Cases (%)	Controls (%)	p
Age at recruitment	< 45 years	214 (19)	234 (18)	< 0.001
	45–54 years	317 (28)	282 (21)	
	55–64 years	296 (26)	290 (22)	
	65–74 years	180 (16)	313 (24)	
	≥ 75 years	132 (12)	203 (15)	
Educational level	Unfinished primary	162 (14)	246 (19)	0.02
	Primary studies	391 (34)	405 (31)	
	Secondarystudies	366 (32)	405 (31)	
	High education	220 (19)	266 (20)	
Parity	Nulliparous	254 (22)	242 (18)	0.007
	1	207 (18)	200 (15)	
	2	445 (39)	516 (39)	
	≥ 3	233 (21)	364 (28)	
Age at menarche	≤ 12 years	436 (38)	487 (37)	0.46
	> 12 years	703 (62)	835 (63)	
Menopausal status	Premenopausal	657 (59)	889 (67)	< 0.001
	Postmenopausal	464 (41)	433 (33)	
Age at menopause	< 50 years	329 (47)	445 (50)	0.18
	≥ 50 years	378 (53)	447 (50)	
Age at first delivery	< 20 years	70 (8)	70 (7)	0.50
	20–24 years	247 (28)	286 (28)	
	25–29 years	329 (37)	410 (40)	
	30–34 years	163 (19)	187 (18)	
	≥ 35 years	69 (8)	64 (6)	
Tobacco use	Non- smoker	607 (53)	792 (60)	0.001
	Former smoker	304 (27)	274 (21)	
	Current smoker	228 (20)	256 (19)	
Body Mass Index (kg/m^2^)	< 18.5	15 (1)	26 (2)	0.34
	18.5–24.9	527 (46)	638 (48)	
	25.0–29.9	387 (34)	414 (31)	
	≥ 30.0	210 (18)	244 (18)	
Family history of breast cancer	No	764 (67)	1097 (83)	< 0.001
	First-degree relative	167 (14)	121 (9)	
	Second-degree relative	24 (2)	14 (1)	
	Other relative	178 (16)	89 (7)	
Hormonal contraceptive	No	628 (55)	725 (55)	0.88
	Yes	511 (45)	597 (45)	
Hormone replacement therapy	No	1021 (90)	1177 (89)	0.70
	Yes	83 (7)	100 (8)	

### Reproductive factors and breast cancer

Table 2 demonstrates the reproductive factors – breast cancer relationship without taking into account genetic information. The number of deliveries shows a dose-response protective effect (OR = 0.58, 95%CI: 0.38–0.90 for women with 1–2 deliveries compared with women with nulliparous, and OR = 0.43, 95% CI: 0.27–0.68 for women with more than 2 deliveries); age at menopause lower than 50 years was also protective (OR = 0.77, 95% CI: 0.62–0.96), while first time delivery when younger than 20 years old increased the risk for breast cancer (OR = 1.65, 95% CI: 1.00–2.75, compared with 25–29 years).

**Table 2:** Relationship between reproductive factors and breast cancer

Reproductive factor	Category	OR (95% CI)	*p* value
Age at menarche	> 12 years	1 (reference)	–
	≤ 12 years	0.95 (0.79–1.13)	0.55
Number of deliveries	0	1 (reference)	–
	1–2	0.58 (0.38–0.90)	0.01
	> 2	0.43 (0.27–0.68)	< 0.001
Age at first delivery^a^	< 20 years	1.65 (1.00–2.75)	0.05
	20–24 years	1.06 (0.83–1.34)	0.65
	25–29 years	1 (reference)	–
	30–34 years	0.93 (0.70–1.23)	0.60
	≥ v35 years	1.11 (0.73–1.69)	0.63
Age at menopause^b^	< 50 years	0.77 (0.62–0.96)	0.02
	≥ 50 years	1 (reference)	–
Use of hormonal contraceptives	No	1 (reference)	–
	Yes	0.81 (0.67–0.97)	0.02
Use of hormone replacement therapy^b^	No	1 (reference)	–
	Yes	1.00 (0.95–1.06)	0.89

Odds ratios (OR) adjusted for propensity score, menopausal status and mutually adjusted for the remaining variables in the table

^a^Odds ratio estimated only in parous women

^b^Odds ratio estimated only in postmenopausal women

### SNP selection

Out of 1314 SNPs preselected, 7 SNPs reached a Cochran-Armitage based *p* value lower than 0.001 and were therefore selected for the main analysis (Additional file 1: Table S1). They were located in 5 genes (RPS6KA1, ATF6B, HSD17B3, CREB3L1 and SRC), which fell under the estrogen signalling pathway (5 genes), the steroid hormone biosynthesis pathway, the GnRH signalling pathway, the progesterone-mediated oocyte maturation pathway, the prolactin signalling pathway, the oxytocin signalling pathway and the prostate cancer signalling pathway (one gene each). The minor allele frequency in controls ranges from 5.5% to 39.7%. Three SNPs (rs204890, rs1269851 and rs204894) were located in the same gene (ATF6B), being 8.4 Kb the longest distance among them; they were in strong linkage disequilibrium with each other (*p* < 0.001), so from here on we limited our analysis to rs1269851 (the one with higher Cochran-Armitage association); results on rs204890 and rs204894 were similar with those on rs1269851.

### Stratified analysis

Table 3 summarizes the stratified analysis on the effect of the number of deliveries and age at menopause; the alleles referred to from here on are the minor alleles in each SNP. Women carrying C allele in rs2229712 (RPS6KA1 gene) did not benefit from the protective effects of number of deliveries, as ORs for 1–2 and > 2 deliveries compared to 0 deliveries were 0.48 (0.28–0.81) and 0.34 (0.19–0.59) in non-carriers, and 0.92 (0.42–1.98) and 0.71 (0.31–1.61) in C carriers. Similar results were found in women carrying the C allele in rs1269851 (ATF6B gene). Carriers of Allele T in rs35652107 (CREB3L1 gene) and allele C in rs6018027 (SRC gene) however, had a more pronounced effect of the number of deliveries, although the *p* values for interaction were not significant in these two cases.

**Table 3:** Relationship between reproductive factors and breast cancer stratifying by genotype

SNP	Genotype	Number of deliveries^a^			Age at menopause^b^	
		0	1–2	> 2	< 50 years	≥ 50 years
All participants		1 (reference)	0.58 (0.38–0.90)	0.43 (0.27–0.68)	0.77 (0.62–0.96)	1 (reference)
rs2229712 (RPS6KA1 gene)	AA genotype (*n* = 1518)	1 (reference)	0.48 (0.28–0.81)	0.34 (0.19–0.59)	0.64 (0.48–0.86)	1 (reference)
	AC or CC genotypes (*n* = 936)	1 (reference)	0.92 (0.42–1.98)	0.71 (0.31–1.61)	1.06 (0.74–1.54)	1 (reference)
rs1269851 (ATF6B gene)	TT genotype (*n* = 2075)	1 (reference)	0.54 (0.34–0.86)	0.38 (0.23–0.62)	0.73 (0.57–0.93)	1 (reference)
	TC or CC genotypes (*n* = 383)	1 (reference)	0.78 (0.24–2.55)	0.69 (0.20–2.42)	1.16 (0.65–2.07)	1 (reference)
rs2026001 (HSD17B3 gene)	GG genotype (*n* = 896)	1 (reference)	0.48 (0.23–0.98)	0.31 (0.14–0.68)	0.73 (0.50–1.06)	1 (reference)
	GT or TT genotypes (*n* = 1559)	1 (reference)	0.64 (0.37–1.09)	0.50 (0.28–0.89)	0.77 (0.58–1.02)	1 (reference)
rs35652107 (CREB3L1 gene)	GG genotype (*n* = 2163)	1 (reference)	0.68 (0.43–1.07)	0.51 (0.31–0.83)	0.73 (0.58–0.93)	1 (reference)
	GT or TT genotypes (*n* = 291)	1 (reference)	0.23 (0.06–0.88)	0.13 (0.03–0.55)	1.20 (0.61–2.39)	1 (reference)
rs6018027 (SRC gene)	TT genotype (*n* = 1251)	1 (reference)	0.86 (0.47–1.58)	0.52 (0.27–1.00)	0.78 (0.57–1.07)	1 (reference)
	CT or CC genotypes (*n* = 1207)	1 (reference)	0.40 (0.21–0.73)	0.35 (0.18–0.67)	0.77 (0.56–1.07)	1 (reference)

^a^Odds ratios and 95% confidence intervals adjusted for propensity score, menopausal status, age at menarche, age at first delivery, age at menopause, use of hormonal contraceptives, use of hormone replacement therapy

^b^odds ratios and 95% confidence intervals adjusted for propensity score, menopausal status, age at menarche, number of deliveries, age at first delivery, use of hormonal contraceptives, use of hormone replacement therapy; estimated in postmenopausal women

The protective effect of the early age at menopause disappeared in women with the C allele in rs2229712 (OR = 0.64, 95% CI: 0.48–0.86 in non-carriers; OR = 1.06, 95% CI: 0.74–1.54 in carriers); the same effect modification was found in women carrying the C allele in rs1269851 and the T allele in rs35652107. No other pattern of effect modification could be found in other reproductive factors. Tables S2-S6 display the effect of reproductive factors on breast cancer risk stratified by genetic variants (See Additional file 1).

To further explore these effect interactions, we constructed an ad hoc genetic score including the four SNPs able to modify the number of deliveries – breast cancer relationship (i.e.: rs2229712, rs1269851, rs35652107 and rs6018027). The genetic score was obtained adding one point each if the minor allele was present in rs2229712 and rs1269851 (where the minor allele carriers did not benefit from the protective effect of the number of deliveries) and one point each if the minor allele was absent in rs35652107 and rs6018027 (where the minor allele carriers had higher protective effect of the number of deliveries). The distribution of this genetic score among cases and controls is summarized in Additional files (See Additional file 1: Table S7). As expected, the protective effect of the number of deliveries disappeared as the genetic score value increased (Table 4).

**Table 4:** Relationship among reproductive factors and breast cancer stratified by the genetic score; odds ratios adjusted for propensity score, menopausal status and the remaining variables in the table

Variable	Category	Genetic score	OR (95% CI)
Number of deliveries	0	Any value	1 (reference)
	1–2	0	0.06 (0.01–0.72)
		1	0.27 (0.12–0.62)
		2	0.82 (0.43–1.59)
		3	0.94 (0.35–2.53)
		4	2.64 (0.09–76.7)
	> 2	0	0.10 (0.01–1.18)
		1	0.24 (0.10–0.58)
		2	0.43 (0.21–0.88)
		3	0.83 (0.29–2.38)
		4	2.90 (0.07–118)
Age at menopause	< 50 years	0	0.93 (0.18–4.69)
		1	0.51 (0.33–0.79)
		2	0.87 (0.62–1.22)
		3	0.95 (0.58–1.55)
		4	1.13 (0.17–7.41)
	≥ 50 years	Any value	1 (reference)

Genetic score obtained adding 1 point each if: rs2229712 C allele is present, rs1269851 C allele is present, rs35652107 A allele is absent, rs6018027 C allele is absent

## Discussion

### Main findings

We found that genetic variants in RPS6KA1, ATF6B, CREB3L1 and SRC genes were associated with modified relationships between reproductive factors and breast cancer, especially the protective effect of the number of deliveries and the early age at menopause. This is the first time, to our knowledge, that genetic variants located in hormonal pathways are found to be modifiers of the reproductive factors – breast cancer relationship. These four genes are involved in hormonal pathways, which suggest a biologically plausible way for this gene-environment interaction.

### Genetic variants in rs1269851 (ATF6B gene) and rs35652107 (CREB3L1 gene)

ATF6B and CREB3L1 are effector proteins of the unfolded protein response (UPR) [10, 11]. When endoplasmic reticulum is exposed to stresses such as hypoxia, glucose depletion or expression of mutant proteins causes the accumulation of unfolded proteins [12]. Trying to counterbalance it, the endoplasmic reticulum activates the UPR [13], which leads to an upturn in the folding capacity of the endoplasmic reticulum and increases the misfolded protein degradation. If this response is unsuccessful, the UPR signals for apoptosis [14]. Proteins’ downstream UPR receptor pathways -such as ATF6B and CREB3L1- have proapoptotic roles [15]. UPR seems to be involved in cancer development, probably by increasing cancer cell resistance to stresses found in the microenvironment [14].

In keeping with our results, mutations in SNPs rs1269851 (ATF6B gene) and rs35652107 (CREB3L1 gene) modify the number of births – breast cancer relationship. In vitro studies have found that mouse granulosa cells with endoplasmic reticulum stress did not show any change in estradiol levels in response to FSH [16]. On the other hand, estrogens can inhibit the apoptosis induced by endoplasmic reticulum stress [17, 18], provoking an interaction with genes regulating UPR such as ATF6B and CREB3L1 possible.

### Genetic variant in rs2229712 (RPS6KA1 gene)

One way for sexual hormones to induce cancer is the ability of estradiol to stimulate BAD phosphorylation and prevent apoptosis, which is mediated via MEK/ERK/RPS6KA1 and PI3K and Akt pathways [19]. Mutations in RPS6KA1 could, therefore, modify the estradiol – breast cancer relationship. In this way, we are reporting that breast cancer risk did not decrease with the number of deliveries in women carrying the allele C in rs2229712. The allele C frequency in the general population (i.e. controls) was as high as 21.5% in our study, implying that as many as 1 out of 5 women would not see their breast cancer risk decreasing with parity. Public health implications of this finding are not clear as the number of deliveries a woman have is not expected to be changed for decreasing breast cancer risk (21). *Genetic variant in rs6018027 (SRC gene).*

SRC gene encodes the protein steroid receptor coactivator-1 (SRC1), which is able to interact with nuclear receptors in the presence of hormones [20]; in particular, the complex SRC1 – estrogen receptor α demonstrates higher affinity binding than other SRC1 – nuclear receptor complexes [21]. Although SRC1 can be regulated via several signaling pathways (Src kinase activity [22], ERK1 and ERK2 -two mitogen-activated protein kinases (MAPK)-[23] or epidermal growth factor (EGF) [24]), our main concern in this paper is the putative pathways for interacting with sexual hormones. In this way, SRC1 is needed for duct elongation in normal mammary tissue during puberty and for secretory alveoli development in pregnancy [24]; which opens a route for modifying the relationship between the number of deliveries and breast cancer.

### Genetic variant in rs2026001 (HSD17B3 gene)

The only gene in our analysis that was involved in steroidogenesis was HSD17B3; we have been unable to find any interaction of this gene with reproductive factors. HSD17B3 encodes 17 beta-hydroxysteroid dehydrogenase isoform 3, whose main function is to catalyze the conversion of androstenedione to testosterone in Leydig cells (testes); testosterone would be peripherally converted to dihydrotestosterone -catalyzed by SRDSA1- and to estradiol -catalyzed by CYP19A1. HSD17B3 gene is also transcribed in both subcutaneous abdominal and omental adipose depots in women [25] although, its relevance for estradiol production remains uncertain.

### Limitations

Several limitations must be mentioned in our results. Firstly, we have selected 7 out of 1314 SNPs by using a Cochran-Armitage 0.001 cut-off *p* value, which is less stringent than the Bonferroni-corrected value of 0.05/1314 ≈ 0.00005. It should be noted, however, that this p value is not used here for testing hypothesis but for screening SNPs before testing them as reproductive factors-effect modifiers. Screening SNPs with cut-off *p* values without multiplicity test adjustment have been used previously for this purpose [26] in GWAS settings.

Secondly, the main assumption in our study was that the reproductive factors effect would be modified by genetic variants via hormonal pathways. The four genes we have identifiedhowever, act in several other pathways (Additional file 1: Table S9), some of them have been recognized as cancer-related. For instance, SRC has a role in 26 pathways, some of them actually related to cancer (hsa04012 [ErbB signaling pathway], hsa05203 [viral carcinogenesis], hsa05205 [proteoglycans in cancer], hsa05219 [bladder cancer]). Thus, we are not attributing its interaction to a specific pathway (the estrogen signalling pathway, for instance). Instead, we are using hormonal pathways as a trail for searching for genes that interact with specific environmental factors, with the specific biological mechanisms considered a matter for lab research.

Thirdly, information on reproductive factors in the MCC-Spain study was self-reported, which makes it more prone to misclassification. However, women participating in this study were not aware of our hypotheses or their genotypes, therefore if some misclassification bias was produced, we can expect it to be non-differential. Fourthly, our study was exploratory by nature: further research is needed to confirm our results.

## Conclusion

In summary, we reported interactions between four genetic variants and reproductive factors in breast cancer risk; this fact, if confirmed, would modify risk scores on breast cancer. As this is the first time such an interaction is reported, caution should be exerted in generalizing our results till further confirmation in independent studies.

## Additional files

Additional file 1: **Table S1.** List of selected SNPs, **Table S2.** Relationship among reproductive factors and breast cancer stratifying by rs2229712 genotype; odds ratios adjusted for propensity score, menopausal status and the remaining variables in the table, **Table S3.** Relationship among reproductive factors and breast cancer stratifying by rs1269851 genotype; odds ratios adjusted for propensity score, menopausal status and the remaining variables in the table, **Table S4.** Relationship between reproductive factors and breast cancer stratified by rs2026001 genotype; odds ratios adjusted for propensity score, menopausal status and the remaining variables in the table, **Table S5.** Relationship between reproductive factors and breast cancer stratified by rs35652107 genotype; odds ratios adjusted for propensity score, menopausal status and the remaining variables in the table, **Table S6.** Relationship between reproductive factors and breast cancer stratified by rs6018027 genotype; odds ratios adjusted for propensity score, menopausal status and the remaining variables in the table, **Table S7.** Distribution of the genetic score, Table S8. List of pathways which the analyzed genes are involved in. (DOCX 43 kb)

## Abbreviations

BMI: Body Mass Index
CI: Confidence intervals
EGF: Epidermal growth factor
GWAS: Genome-wide association studies
KEGG: Kyoto Encyclopaedia of genes and genomes
MAPK: Mitogen-Activated protein kinases
OR: Odds ratios
SRC1: Steroid receptor coactivator-1
UPR: Unfolded protein response
