# Supplementary material for: Reproductive risk factors in breast cancer and genetic hormonal pathways: a gene-environment interaction in the MCC-Spain project
Source: BMC Cancer. 2018 Mar 12;18:280. doi: 10.1186/s12885-018-4182-3 (PMC5848450; doi:10.1186/s12885-018-4182-3)
Supplement: Supplementary file 1 — Table S1. List of selected SNPs, Table S2. Relationship among reproductive factors and breast cancer stratifying by rs2229712 genotype; odds ratios adjusted for propensity score, menopausal status and the remaining variables in the table, Table S3. Relationship among reproductive factors and breast cancer stratifying by rs1269851 genotype; odds ratios adjusted for propensity score, menopausal status and the remaining variables in the table, Table S4. Relationship between reproductive factors and breast cancer stratified by rs2026001 genotype; odds ratios adjusted for propensity score, menopausal status and the remaining variables in the table, Table S5. Relationship between reproductive factors and breast cancer stratified by rs35652107 genotype; odds ratios adjusted for propensity score, menopausal status and the remaining variables in the table, Table S6. Relationship between reproductive factors and breast cancer stratified by rs6018027 genotype; odds ratios adjusted for propensity score, menopausal status and the remaining variables in the table, Table S7. Distribution of the genetic score, Table S8. List of pathways which the analyzed genes are involved in. (DOCX 43 kb) [file 12885_2018_4182_MOESM1_ESM.docx]

Additional file 1 Table S1. List of selected SNPs

| SNP | Chromosome | Position | Gene | Alleles | MAF | Keggs hormonal pathways | Cochran-Armitage based p value |
| --- | --- | --- | --- | --- | --- | --- | --- |
| rs2229712 | 1 | 26883511 | RPS6KA1 | A/C | 21.5 | hsa04914 | 0.0003 |
| rs204890 | 6 | 32085598 | ATF6B | C/T | 8.3 | hsa04915 | 0.0001 |
| rs1269851 | 6 | 32092207 | ATF6B | T/C | 8.4 | hsa04915 | 0.00005 |
| rs204894 | 6 | 32093922 | ATF6B | G/A | 8.8 | hsa04915 | 0.0003 |
| rs2026001 | 9 | 99016095 | HSD17B3 | G/T | 39.7 | hsa00140 | 0.0003 |
| rs35652107 | 11 | 46339011 | CREB3L1 | G/A | 5.5 | hsa04915  hsa05215 | 0.0003 |
| rs6018027 | 20 | 35990792 | SRC | T/C | 28.5 | hsa04912  hsa04915  hsa04917  hsa04921 | 0.0005 |

MAF: Minor allele frequency in our control sample. Pathways: hsa00140: Steroid hormone biosynthesis; hs4912: GnRH signaling pathway; hsa04914: Progesterone-mediated oocyte maturation; hsa04915: Estrogen signaling pathway; hsa04917: Prolactin signaling pathway; hsa04921: Oxytocin signaling pathway; hsa05215: Prostate cancer

Table S2. Relationship among reproductive factors and breast cancer stratifying by rs2229712 genotype; odds ratios adjusted for propensity score, menopausal status and the remaining variables in the table

| Reproductive factor | Category | OR (95% CI) in patients with rs2229712 AA genotype (n=1518) | OR (95% CI) in patients with rs2229712 AC or CC genotypes (n=936) | p for interaction |
| --- | --- | --- | --- | --- |
| Age at menarche | >12 years | 1 (reference) | 1 (reference) |  |
|  | ≤12 years | 1.03 (0.82 – 1.29) | 0.81 (0.61 – 1.09) | 0.43 |
| Number of deliveries | 0 | 1 (reference) | 1 (reference) |  |
|  | 1-2 | 0.48 (0.28 – 0.81) | 0.92 (0.42 – 1.98) | 0.03 |
|  | >2 | 0.34 (0.19 – 0.59) | 0.71 (0.31 – 1.61) | 0.007 |
| Age at first delivery* | <20 years | 1.36 (0.71 – 2.61) | 2.21 (0.96 – 5.11) | 0.77 |
|  | 20-24 years | 1.08 (0.79 – 1.47) | 0.98 (0.67 – 1.44) | 0.63 |
|  | 25-29 years | 1 (reference) | 1 (reference) |  |
|  | 30-34 years | 0.87 (0.61 – 1.26) | 0.93 (0.59 – 1.46) | 0.67 |
|  | ≥35 years | 1.04 (0.61 – 1.77) | 1.21 (0.59 – 2.48) | 0.84 |
| Age at menopause** | <50 years | 0.64 (0.48 – 0.86) | 1.06 (0.74 – 1.54) | 0.005 |
|  | ≥50 years | 1 (reference) | 1 (reference) |  |
| Ever use of hormonal contraceptives | No | 1 (reference) | 1 (reference) |  |
|  | Yes | 0.72 (0.57 – 0.91) | 0.99 (0.74 – 1.33) | 0.01 |
| Ever use of hormone replacement therapy** | No | 1 (reference) | 1 (reference) |  |
|  | Yes | 1.00 (0.93 – 1.07) | 1.02 (0.93 – 1.12) | 0.22 |

*Odds ratio estimated only in parous women. **Odds ratio estimated only in postmenopausal women.

Table S3. Relationship among reproductive factors and breast cancer stratifying by rs1269851 genotype; odds ratios adjusted for propensity score, menopausal status and the remaining variables in the table

| Reproductive factor | Category | OR (95% CI) in patients with rs1269851 TT genotype (n=2075) | OR (95% CI) in patients with rs1269851 TC or CC genotypes (n=383) | p for interaction |
| --- | --- | --- | --- | --- |
| Age at menarche | >12 years | 1 (reference) | 1 (reference) |  |
|  | ≤12 years | 0.96 (0.79 – 1.16) | 0.88 (0.57 – 1.39) | 0.98 |
| Number of deliveries | 0 | 1 (reference) | 1 (reference) |  |
|  | 1-2 | 0.54 (0.34 – 0.86) | 0.78 (0.24 – 2.55) | 0.32 |
|  | >2 | 0.38 (0.23 – 0.62) | 0.69 (0.20 – 2.42) | 0.06 |
| Age at first delivery* | <20 years | 1.83 (1.05 – 3.17) | 0.58 (0.11 – 3.08) | 0.09 |
|  | 20-24 years | 0.96 (0.74 – 1.25) | 1.74 (0.96 – 3.16) | 0.10 |
|  | 25-29 years | 1 (reference) | 1 (reference) |  |
|  | 30-34 years | 0.89 (0.65 – 1.20) | 1.14 (0.53 – 2.44) | 0.56 |
|  | ≥35 years | 1.04 (0.65 – 1.66) | 1.61 (0.61 – 4.25) | 0.66 |
| Age at menopause** | <50 years | 0.73 (0.57 – 0.93) | 1.16 (0.65 – 2.07) | 0.07 |
|  | ≥50 years | 1 (reference) | 1 (reference) |  |
| Use of hormonal contraceptives | No | 1 (reference) | 1 (reference) |  |
|  | Yes | 0.82 (0.67 – 1.01) | 0.71 (0.44 – 1.13) | 0.85 |
| Use of hormone replacement therapy** | No | 1 (reference) | 1 (reference) |  |
|  | Yes | 1.01 (0.95 – 1.08) | 0.97 (0.83 – 1.12) | 0.39 |

*Odds ratio estimated only in parous women. **Odds ratio estimated only in postmenopausal women.

Table S4. Relationship between reproductive factors and breast cancer stratified by rs2026001 genotype; odds ratios adjusted for propensity score, menopausal status and the remaining variables in the table

| Reproductive factor | Category | OR (95% CI) in patients with rs2026001 GG genotype (n=896) | OR (95% CI) in patients with rs2026001 GT or TT genotypes (n=1559) | p for interaction |
| --- | --- | --- | --- | --- |
| Age at menarche | >12 years | 1 (reference) | 1 (reference) |  |
|  | ≤12 years | 1.00 (0.75 – 1.34) | 0.91 (0.73 – 1.14) | 0.81 |
| Number of deliveries | 0 | 1 (reference) | 1 (reference) |  |
|  | 1-2 | 0.48 (0.23 – 0.98) | 0.64 (0.37 – 1.09) | 0.05 |
|  | >2 | 0.31 (0.14 – 0.68) | 0.50 (0.28 – 0.89) | 0.002 |
| Age at first delivery* | <20 years | 1.70 (0.81 – 3.56) | 1.65 (0.81 – 3.34) | 0.46 |
|  | 20-24 years | 1.15 (0.76 – 1.73) | 1.03 80.76 – 1.39) | 0.74 |
|  | 25-29 years | 1 (reference) | 1 (reference) |  |
|  | 30-34 years | 1.15 (0.72 – 1.81) | 0.84 (0.59 – 1.21) | 0.32 |
|  | ≥35 years | 1.39 (0.71 – 2.72) | 1.00 (0.59 – 1.71) | 0.27 |
| Age at menopause** | <50 years | 0.73 (0.50 – 1.06) | 0.77 (0.58 – 1.02) | 0.52 |
|  | ≥50 years | 1 (reference) | 1 (reference) |  |
| Use of hormonal contraceptives | No | 1 (reference) | 1 (reference) |  |
|  | Yes | 0.74 (0.55 – 1.01) | 0.84 (0.62 – 1.07) | 0.25 |
| Use of hormone replacement therapy** | No | 1 (reference) | 1 (reference) |  |
|  | Yes | 0.97 (0.89 – 1.06) | 1.04 (0.96 – 1.12) | 0.05 |

*Odds ratio estimated only in parous women. **Odds ratio estimated only in postmenopausal women.

Table S5. Relationship between reproductive factors and breast cancer stratified by rs35652107 genotype; odds ratios adjusted for propensity score, menopausal status and the remaining variables in the table

| Reproductive factor | Category | OR (95% CI) in patients with rs35652107 GG genotype (n=2163) | OR (95% CI) in patients with rs35652107 GT or TT genotypes (n=291) | p for interaction |
| --- | --- | --- | --- | --- |
| Age at menarche | >12 years | 1 (reference) | 1 (reference) |  |
|  | ≤12 years | 0.93 (0.77 – 1.12) | 1.13 (0.66 – 1.95) | 0.42 |
| Number of deliveries | 0 | 1 (reference) | 1 (reference) |  |
|  | 1-2 | 0.68 (0.43 – 1.07) | 0.23 (0.06 – 0.88) | 0.63 |
|  | >2 | 0.51 (0.31 – 0.83) | 0.13 (0.03 – 0.55) | 0.67 |
| Age at first delivery* | <20 years | 1.63 (0.95 – 2.79) | 1.43 (0.29 – 7.15) | 0.48 |
|  | 20-24 years | 0.96 (0.74 – 1.24) | 2.16 (1.05 – 4.43) | 0.05 |
|  | 25-29 years | 1 (reference) | 1 (reference) |  |
|  | 30-34 years | 0.86 (0.64 – 1.16) | 1.81 (0.72 – 4.51) | 0.07 |
|  | ≥35 years | 1.13 (0.72 – 1.78) | 0.70 (0.20 – 2.43) | 0.49 |
| Age at menopause** | <50 years | 0.73 (0.58 – 0.93) | 1.20 (0.61 – 2.39) | 0.06 |
|  | ≥50 years | 1 (reference) | 1 (reference) |  |
| Use of hormonal contraceptives | No | 1 (reference) | 1 (reference) |  |
|  | Yes | 0.82 (0.67 – 1.00) | 0.72 (0.41 – 1.27) | 0.41 |
| Use of hormone replacement therapy** | No | 1 (reference) | 1 (reference) |  |
|  | Yes | 1.00 (0.94 – 1.07) | 1.01 (0.85 – 1.21) | 0.55 |

*Odds ratio estimated only in parous women. **Odds ratio estimated only in postmenopausal women.

Table S6. Relationship between reproductive factors and breast cancer stratified by rs6018027 genotype; odds ratios adjusted for propensity score, menopausal status and the remaining variables in the table

| Reproductive factor | Category | OR (95% CI) in patients with rs6018027 TT genotype (n=1251) | OR (95% CI) in patients with rs6018027 CT or CC genotypes (n=1207) | p for interaction |
| --- | --- | --- | --- | --- |
| Age at menarche | >12 years | 1 (reference) | 1 (reference) |  |
|  | ≤12 years | 0.89 (0.70 – 1.15) | 1.03 (0.80 – 1.33) | 0.32 |
| Number of deliveries | 0 | 1 (reference) | 1 (reference) |  |
|  | 1-2 | 0.86 (0.47 – 1.58) | 0.40 (0.21 – 0.73) | 0.37 |
|  | >2 | 0.52 (0.27 – 1.00) | 0.35 (0.18 – 0.67) | 0.29 |
| Age at first delivery* | <20 years | 2.17 (1.00 – 4.67) | 1.33 (0.67 – 2.64) | 0.22 |
|  | 20-24 years | 1.05 (0.75 – 1.47) | 1.08 (0.76 – 1.52) | 0.96 |
|  | 25-29 years | 1 (reference) | 1 (reference) |  |
|  | 30-34 years | 0.87 (0.60 – 1.27) | 1.02 (0.66 – 1.56) | 0.48 |
|  | ≥35 years | 1.65 (0.90 – 3.02) | 0.78 (0.43 – 1.43) | 0.05 |
| Age at menopause** | <50 years | 0.78 (0.57 – 1.07) | 0.77 (0.56 – 1.07) | 0.38 |
|  | ≥50 years | 1 (reference) | 1 (reference) |  |
| Use of hormonal contraceptives | No | 1 (reference) | 1 (reference) |  |
|  | Yes | 0.77 (0.58 – 1.04) | 0.85 (0.66 – 1.11) | 0.24 |
| Use of hormone replacement therapy** | No | 1 (reference) | 1 (reference) |  |
|  | Yes | 0.99 (0.92 – 1.07) | 1.01 (0.93 – 1.10) | 0.56 |

*Odds ratio estimated only in parous women. **Odds ratio estimated only in postmenopausal women.

Table S7. Distribution of the genetic score

| Genetic score | Cases (%) | Controls (%) | p value |
| --- | --- | --- | --- |
| 0 | 37 (3) | 45 (3) | 0.60 |
| 1 | 331 (29) | 348 (26) |  |
| 2 | 505 (44) | 600 (45) |  |
| 3 | 236 (21) | 293 (22) |  |
| 4 | 27 (2) | 36 (3) |  |

Genetic score obtained adding 1 point each if: rs2229712 C allele is present, rs1269851 C allele is present, rs35652107 A allele is absent, rs6018027 C allele is absent.

Table S8. List of pathways which the analyzed genes are involved in

| **Gene** | **Pathway code (KEGG)** | **Pathway name** |
| --- | --- | --- |
| ATF6B | [hsa04022](http://www.kegg.jp/pathway/hsa04022) | [· cGMP-PKG signaling pathway, organism-specific biosystem](http://www.ncbi.nlm.nih.gov/biosystems/983748?Sel=geneid:1388#show=genes) |
| ATF6B | [hsa04141](http://www.kegg.jp/pathway/hsa04141) | [· Protein processing in endoplasmic reticulum, organism-specific biosystem](http://www.ncbi.nlm.nih.gov/biosystems/167325?Sel=geneid:1388#show=genes) |
| ATF6B | [hsa04151](http://www.kegg.jp/pathway/hsa04151) | [· PI3K-Akt signaling pathway, organism-specific biosystem](http://www.ncbi.nlm.nih.gov/biosystems/692234?Sel=geneid:1388#show=genes) |
| ATF6B | [hsa04211](http://www.kegg.jp/pathway/hsa04211) | [· Longevity regulating pathway, organism-specific biosystem](http://www.ncbi.nlm.nih.gov/biosystems/1319989?Sel=geneid:1388#show=genes) |
| ATF6B | [hsa04261](http://www.kegg.jp/pathway/hsa04261) | [· Adrenergic signaling in cardiomyocytes, organism-specific biosystem](http://www.ncbi.nlm.nih.gov/biosystems/908257?Sel=geneid:1388#show=genes) |
| ATF6B | [hsa04668](http://www.kegg.jp/pathway/hsa04668) | [· TNF signaling pathway, organism-specific biosystem](http://www.ncbi.nlm.nih.gov/biosystems/812256?Sel=geneid:1388#show=genes) |
| ATF6B | [hsa04728](http://www.kegg.jp/pathway/hsa04728) | [· Dopaminergic synapse, organism-specific biosystem](http://www.ncbi.nlm.nih.gov/biosystems/469199?Sel=geneid:1388#show=genes) |
| ATF6B | [hsa04911](http://www.kegg.jp/pathway/hsa04911) | [· Insulin secretion, organism-specific biosystem](http://www.ncbi.nlm.nih.gov/biosystems/777534?Sel=geneid:1388#show=genes) |
| ATF6B | [hsa04915](http://www.kegg.jp/pathway/hsa04915) | [· Estrogen signaling pathway, organism-specific biosystem](http://www.ncbi.nlm.nih.gov/biosystems/799177?Sel=geneid:1388#show=genes) |
| ATF6B | [hsa04918](http://www.kegg.jp/pathway/hsa04918) | [· Thyroid hormone synthesis, organism-specific biosystem](http://www.ncbi.nlm.nih.gov/biosystems/835410?Sel=geneid:1388#show=genes) |
| ATF6B | [hsa04925](http://www.kegg.jp/pathway/hsa04925) | [· Aldosterone synthesis and secretion, organism-specific biosystem](http://www.ncbi.nlm.nih.gov/biosystems/1272485?Sel=geneid:1388#show=genes) |
| ATF6B | [hsa05030](http://www.kegg.jp/pathway/hsa05030) | [· Cocaine addiction, organism-specific biosystem](http://www.ncbi.nlm.nih.gov/biosystems/546258?Sel=geneid:1388#show=genes) |
| ATF6B | [hsa05031](http://www.kegg.jp/pathway/hsa05031) | [· Amphetamine addiction, organism-specific biosystem](http://www.ncbi.nlm.nih.gov/biosystems/547607?Sel=geneid:1388#show=genes) |
| ATF6B | [hsa05034](http://www.kegg.jp/pathway/hsa05034) | [· Alcoholism, organism-specific biosystem](http://www.ncbi.nlm.nih.gov/biosystems/585563?Sel=geneid:1388#show=genes) |
| ATF6B | [hsa05161](http://www.kegg.jp/pathway/hsa05161) | [· Hepatitis B, organism-specific biosystem](http://www.ncbi.nlm.nih.gov/biosystems/694606?Sel=geneid:1388#show=genes) |
| ATF6B | [hsa05203](http://www.kegg.jp/pathway/hsa05203) | [· Viral carcinogenesis, organism-specific biosystem](http://www.ncbi.nlm.nih.gov/biosystems/658418?Sel=geneid:1388#show=genes) |
|  |  |  |
| CREB3L1 | [hsa04022](http://www.kegg.jp/pathway/hsa04022) | [· cGMP-PKG signaling pathway, organism-specific biosystem](http://www.ncbi.nlm.nih.gov/biosystems/983748?Sel=geneid:90993#show=genes) |
| CREB3L1 | [hsa04024](http://www.kegg.jp/pathway/hsa04024) | [· cAMP signaling pathway, organism-specific biosystem](http://www.ncbi.nlm.nih.gov/biosystems/1017634?Sel=geneid:90993#show=genes) |
| CREB3L1 | [hsa04151](http://www.kegg.jp/pathway/hsa04151) | [· PI3K-Akt signaling pathway, organism-specific biosystem](http://www.ncbi.nlm.nih.gov/biosystems/692234?Sel=geneid:90993#show=genes) |
| CREB3L1 | [hsa04152](http://www.kegg.jp/pathway/hsa04152) | [· AMPK signaling pathway, organism-specific biosystem](http://www.ncbi.nlm.nih.gov/biosystems/989139?Sel=geneid:90993#show=genes) |
| CREB3L1 | [hsa04211](http://www.kegg.jp/pathway/hsa04211) | [· Longevity regulating pathway, organism-specific biosystem](http://www.ncbi.nlm.nih.gov/biosystems/1319989?Sel=geneid:90993#show=genes) |
| CREB3L1 | [hsa04261](http://www.kegg.jp/pathway/hsa04261) | [· Adrenergic signaling in cardiomyocytes, organism-specific biosystem](http://www.ncbi.nlm.nih.gov/biosystems/908257?Sel=geneid:90993#show=genes) |
| CREB3L1 | [hsa04668](http://www.kegg.jp/pathway/hsa04668) | [· TNF signaling pathway, organism-specific biosystem](http://www.ncbi.nlm.nih.gov/biosystems/812256?Sel=geneid:90993#show=genes) |
| CREB3L1 | [hsa04725](http://www.kegg.jp/pathway/hsa04725) | [· Cholinergic synapse, organism-specific biosystem](http://www.ncbi.nlm.nih.gov/biosystems/217716?Sel=geneid:90993#show=genes) |
| CREB3L1 | [hsa04728](http://www.kegg.jp/pathway/hsa04728) | [· Dopaminergic synapse, organism-specific biosystem](http://www.ncbi.nlm.nih.gov/biosystems/469199?Sel=geneid:90993#show=genes) |
| CREB3L1 | [hsa04915](http://www.kegg.jp/pathway/hsa04915) | [· Estrogen signaling pathway, organism-specific biosystem](http://www.ncbi.nlm.nih.gov/biosystems/799177?Sel=geneid:90993#show=genes) |
| CREB3L1 | [hsa04916](http://www.kegg.jp/pathway/hsa04916) | [· Melanogenesis, organism-specific biosystem](http://www.ncbi.nlm.nih.gov/biosystems/83092?Sel=geneid:90993#show=genes) |
| CREB3L1 | [hsa04918](http://www.kegg.jp/pathway/hsa04918) | [· Thyroid hormone synthesis, organism-specific biosystem](http://www.ncbi.nlm.nih.gov/biosystems/835410?Sel=geneid:90993#show=genes) |
| CREB3L1 | [hsa04922](http://www.kegg.jp/pathway/hsa04922) | [· Glucagon signaling pathway, organism-specific biosystem](http://www.ncbi.nlm.nih.gov/biosystems/1144996?Sel=geneid:90993#show=genes) |
| CREB3L1 | [hsa04925](http://www.kegg.jp/pathway/hsa04925) | [· Aldosterone synthesis and secretion, organism-specific biosystem](http://www.ncbi.nlm.nih.gov/biosystems/1272485?Sel=geneid:90993#show=genes) |
| CREB3L1 | [hsa04931](http://www.kegg.jp/pathway/hsa04931) | [· Insulin resistance, organism-specific biosystem](http://www.ncbi.nlm.nih.gov/biosystems/1272486?Sel=geneid:90993#show=genes) |
| CREB3L1 | [hsa04962](http://www.kegg.jp/pathway/hsa04962) | [· Vasopressin-regulated water reabsorption, organism-specific biosystem](http://www.ncbi.nlm.nih.gov/biosystems/143700?Sel=geneid:90993#show=genes) |
| CREB3L1 | [hsa05016](http://www.kegg.jp/pathway/hsa05016) | [· Huntington's disease, organism-specific biosystem](http://www.ncbi.nlm.nih.gov/biosystems/83100?Sel=geneid:90993#show=genes) |
| CREB3L1 | [hsa05030](http://www.kegg.jp/pathway/hsa05030) | [· Cocaine addiction, organism-specific biosystem](http://www.ncbi.nlm.nih.gov/biosystems/546258?Sel=geneid:90993#show=genes) |
| CREB3L1 | [hsa05031](http://www.kegg.jp/pathway/hsa05031) | [· Amphetamine addiction, organism-specific biosystem](http://www.ncbi.nlm.nih.gov/biosystems/547607?Sel=geneid:90993#show=genes) |
| CREB3L1 | [hsa05034](http://www.kegg.jp/pathway/hsa05034) | [· Alcoholism, organism-specific biosystem](http://www.ncbi.nlm.nih.gov/biosystems/585563?Sel=geneid:90993#show=genes) |
| CREB3L1 | [hsa05161](http://www.kegg.jp/pathway/hsa05161) | [· Hepatitis B, organism-specific biosystem](http://www.ncbi.nlm.nih.gov/biosystems/694606?Sel=geneid:90993#show=genes) |
| CREB3L1 | [hsa05203](http://www.kegg.jp/pathway/hsa05203) | [· Viral carcinogenesis, organism-specific biosystem](http://www.ncbi.nlm.nih.gov/biosystems/658418?Sel=geneid:90993#show=genes) |
| CREB3L1 | [hsa05215](http://www.kegg.jp/pathway/hsa05215) | [· Prostate cancer, organism-specific biosystem](http://www.ncbi.nlm.nih.gov/biosystems/83111?Sel=geneid:90993#show=genes) |
|  |  |  |
| HSD17B3 | [hsa00140](http://www.kegg.jp/pathway/hsa00140) | [· Steroid hormone biosynthesis, organism-specific biosystem](http://www.ncbi.nlm.nih.gov/biosystems/82940?Sel=geneid:3293#show=genes) |
| HSD17B3 | [hsa01100](http://www.kegg.jp/pathway/hsa01100) | [· Metabolic pathways, organism-specific biosystem](http://www.ncbi.nlm.nih.gov/biosystems/132956?Sel=geneid:3293#show=genes) |
|  |  |  |
| RPS6KA1 | [hsa04010](http://www.kegg.jp/pathway/hsa04010) | [· MAPK signaling pathway, organism-specific biosystem](http://www.ncbi.nlm.nih.gov/biosystems/83048?Sel=geneid:6195#show=genes) |
| RPS6KA1 | [hsa04114](http://www.kegg.jp/pathway/hsa04114) | [· Oocyte meiosis, organism-specific biosystem](http://www.ncbi.nlm.nih.gov/biosystems/126909?Sel=geneid:6195#show=genes) |
| RPS6KA1 | [hsa04150](http://www.kegg.jp/pathway/hsa04150) | [· mTOR signaling pathway, organism-specific biosystem](http://www.ncbi.nlm.nih.gov/biosystems/83059?Sel=geneid:6195#show=genes) |
| RPS6KA1 | [hsa04720](http://www.kegg.jp/pathway/hsa04720) | [· Long-term potentiation, organism-specific biosystem](http://www.ncbi.nlm.nih.gov/biosystems/83085?Sel=geneid:6195#show=genes) |
| RPS6KA1 | [hsa04722](http://www.kegg.jp/pathway/hsa04722) | [· Neurotrophin signaling pathway, organism-specific biosystem](http://www.ncbi.nlm.nih.gov/biosystems/101143?Sel=geneid:6195#show=genes) |
| RPS6KA1 | [hsa04914](http://www.kegg.jp/pathway/hsa04914) | [· Progesterone-mediated oocyte maturation, organism-specific biosystem](http://www.ncbi.nlm.nih.gov/biosystems/119304?Sel=geneid:6195#show=genes) |
| RPS6KA1 | [hsa04931](http://www.kegg.jp/pathway/hsa04931) | [· Insulin resistance, organism-specific biosystem](http://www.ncbi.nlm.nih.gov/biosystems/1272486?Sel=geneid:6195#show=genes) |
|  |  |  |
| SRC | [hsa04012](http://www.kegg.jp/pathway/hsa04012) | [· ErbB signaling pathway, organism-specific biosystem](http://www.ncbi.nlm.nih.gov/biosystems/83049?Sel=geneid:6714#show=genes) |
| SRC | [hsa04015](http://www.kegg.jp/pathway/hsa04015) | [· Rap1 signaling pathway, organism-specific biosystem](http://www.ncbi.nlm.nih.gov/biosystems/868086?Sel=geneid:6714#show=genes) |
| SRC | [hsa04062](http://www.kegg.jp/pathway/hsa04062) | [· Chemokine signaling pathway, organism-specific biosystem](http://www.ncbi.nlm.nih.gov/biosystems/99051?Sel=geneid:6714#show=genes) |
| SRC | [hsa04144](http://www.kegg.jp/pathway/hsa04144) | [· Endocytosis, organism-specific biosystem](http://www.ncbi.nlm.nih.gov/biosystems/102279?Sel=geneid:6714#show=genes) |
| SRC | [hsa04360](http://www.kegg.jp/pathway/hsa04360) | [· Axon guidance, organism-specific biosystem](http://www.ncbi.nlm.nih.gov/biosystems/83065?Sel=geneid:6714#show=genes) |
| SRC | [hsa04370](http://www.kegg.jp/pathway/hsa04370) | [· VEGF signaling pathway, organism-specific biosystem](http://www.ncbi.nlm.nih.gov/biosystems/83066?Sel=geneid:6714#show=genes) |
| SRC | [hsa04510](http://www.kegg.jp/pathway/hsa04510) | [· Focal adhesion, organism-specific biosystem](http://www.ncbi.nlm.nih.gov/biosystems/83067?Sel=geneid:6714#show=genes) |
| SRC | [hsa04520](http://www.kegg.jp/pathway/hsa04520) | [· Adherens junction, organism-specific biosystem](http://www.ncbi.nlm.nih.gov/biosystems/83070?Sel=geneid:6714#show=genes) |
| SRC | [hsa04530](http://www.kegg.jp/pathway/hsa04530) | [· Tight junction, organism-specific biosystem](http://www.ncbi.nlm.nih.gov/biosystems/83071?Sel=geneid:6714#show=genes) |
| SRC | [hsa04540](http://www.kegg.jp/pathway/hsa04540) | [· Gap junction, organism-specific biosystem](http://www.ncbi.nlm.nih.gov/biosystems/83072?Sel=geneid:6714#show=genes) |
| SRC | [hsa04611](http://www.kegg.jp/pathway/hsa04611) | [· Platelet activation, organism-specific biosystem](http://www.ncbi.nlm.nih.gov/biosystems/952858?Sel=geneid:6714#show=genes) |
| SRC | [hsa04727](http://www.kegg.jp/pathway/hsa04727) | [· GABAergic synapse, organism-specific biosystem](http://www.ncbi.nlm.nih.gov/biosystems/377263?Sel=geneid:6714#show=genes) |
| SRC | [hsa04750](http://www.kegg.jp/pathway/hsa04750) | [· Inflammatory mediator regulation of TRP channels, organism-specific biosystem](http://www.ncbi.nlm.nih.gov/biosystems/948277?Sel=geneid:6714#show=genes) |
| SRC | [hsa04810](http://www.kegg.jp/pathway/hsa04810) | [· Regulation of actin cytoskeleton, organism-specific biosystem](http://www.ncbi.nlm.nih.gov/biosystems/83089?Sel=geneid:6714#show=genes) |
| SRC | [hsa04912](http://www.kegg.jp/pathway/hsa04912) | [· GnRH signaling pathway, organism-specific biosystem](http://www.ncbi.nlm.nih.gov/biosystems/83091?Sel=geneid:6714#show=genes) |
| SRC | [hsa04915](http://www.kegg.jp/pathway/hsa04915) | [· Estrogen signaling pathway, organism-specific biosystem](http://www.ncbi.nlm.nih.gov/biosystems/799177?Sel=geneid:6714#show=genes) |
| SRC | [hsa04917](http://www.kegg.jp/pathway/hsa04917) | [· Prolactin signaling pathway, organism-specific biosystem](http://www.ncbi.nlm.nih.gov/biosystems/814182?Sel=geneid:6714#show=genes) |
| SRC | [hsa04921](http://www.kegg.jp/pathway/hsa04921) | [· Oxytocin signaling pathway, organism-specific biosystem](http://www.ncbi.nlm.nih.gov/biosystems/952859?Sel=geneid:6714#show=genes) |
| SRC | [hsa05100](http://www.kegg.jp/pathway/hsa05100) | [· Bacterial invasion of epithelial cells, organism-specific biosystem](http://www.ncbi.nlm.nih.gov/biosystems/149807?Sel=geneid:6714#show=genes) |
| SRC | [hsa05120](http://www.kegg.jp/pathway/hsa05120) | [· Epithelial cell signaling in Helicobacter pylori infection, organism-specific biosystem](http://www.ncbi.nlm.nih.gov/biosystems/83102?Sel=geneid:6714#show=genes) |
| SRC | [hsa05131](http://www.kegg.jp/pathway/hsa05131) | [· Shigellosis, organism-specific biosystem](http://www.ncbi.nlm.nih.gov/biosystems/83104?Sel=geneid:6714#show=genes) |
| SRC | [hsa05152](http://www.kegg.jp/pathway/hsa05152) | [· Tuberculosis, organism-specific biosystem](http://www.ncbi.nlm.nih.gov/biosystems/213780?Sel=geneid:6714#show=genes) |
| SRC | [hsa05161](http://www.kegg.jp/pathway/hsa05161) | [· Hepatitis B, organism-specific biosystem](http://www.ncbi.nlm.nih.gov/biosystems/694606?Sel=geneid:6714#show=genes) |
| SRC | [hsa05203](http://www.kegg.jp/pathway/hsa05203) | [· Viral carcinogenesis, organism-specific biosystem](http://www.ncbi.nlm.nih.gov/biosystems/658418?Sel=geneid:6714#show=genes) |
| SRC | [hsa05205](http://www.kegg.jp/pathway/hsa05205) | [· Proteoglycans in cancer, organism-specific biosystem](http://www.ncbi.nlm.nih.gov/biosystems/782000?Sel=geneid:6714#show=genes) |
| SRC | [hsa05219](http://www.kegg.jp/pathway/hsa05219) | [· Bladder cancer, organism-specific biosystem](http://www.ncbi.nlm.nih.gov/biosystems/83115?Sel=geneid:6714#show=genes) |
